# Airborne infection control in healthcare settings

**DOI:** 10.3402/iee.v3i0.21411

**Published:** 2013-06-14

**Authors:** Saurabh R. Shrivastava, Prateek S. Shrivastava, Jegadeesh Ramasamy

**Affiliations:** Department of Community Medicine, Shri Sathya Sai Medical College and Research Institute, Kancheepuram, India

**Keywords:** airborne, infection, air change, nosocomial

## Abstract

Exposure of human beings to different airborne pathogens has resulted in the emergence of epidemics of respiratory infections. This airborne transmission becomes even more prevalent in healthcare settings because of overburdened hospitals and the presence of immunosuppressed patients. Airborne infection control measures include a three-pronged approach, namely administrative control, environmental control, and personal respiratory protection measures. To conclude, need of the hour is to assess the true contribution of airborne transmission to infection rates so that hospital administrators can implement appropriate control measures to keep respiratory infections under check.

Exposure of human beings to different airborne pathogens has resulted in the emergence of epidemics of respiratory infections (1). Most of the microorganisms released from infectious patients can disperse in a wide geographical area by air currents and finally can be inhaled by susceptible individuals who have had no direct contact with the primary source (2). This airborne transmission becomes even more prevalent in healthcare settings because of overburdened hospitals and the presence of immunosuppressed patients (3).

World Health Organization, Center for Disease Control and Prevention, and International Union of Tuberculosis and Lung Disease have released guidelines regarding the control of airborne infection in both developed and developing nations (4). The suggested measures include a three-pronged approach, namely administrative control, environmental control, and personal respiratory protection measures (4, 5).

The rationale for implementing administrative control measures is to minimize potential opportunities of exposure of susceptible individuals to infectious patients. Administrative control measures include education and training of staff (i.e., principles and practices of infection control/clear roles and responsibilities of different cadres of healthcare staff); outpatient department measures (i.e., screening of patients for respiratory complaints/information – education – communication for promoting cough etiquette by the patients/segregation of respiratory symptomatic in a separate well – ventilated waiting area/fast – tracking of respiratory symptomatic/seating arrangement of doctor and patients); and inpatient department measures (i.e., minimizing hospitalization of patients/reduction of nosocomial infections/educating patients and attendants about cough hygiene/routine segregation of patients to separate infectious wards or separate areas in same ward/maintain spacing by ward decompression/advocating safe sputum collection) (4, 5).

Environmental control measures are recommended mainly for reduction in concentration of infectious particles, which are usually present in the healthcare settings. It comprises indoor patient segregation and bed spacing; ensuring effective ventilation at all times and in all seasons; and giving special attention to high-risk areas, such as anti-retroviral therapy centers, outpatient and inpatient departments, bronchoscopy procedure rooms, and multidrug resistant tuberculosis wards. Strategies like natural ventilation, mechanical ventilation, air changes per hour (a measure of how many times the air within a defined space is replaced per hour), and upper room ultraviolet light have been proposed to ensure adequate ventilation (4, 6).

By promoting personal respiratory protection among the patients and healthcare staff, there is a reduction in risk to other staff and patients. Finding of a study has revealed that multi-sectoral approach should be advocated instead of an individual approach for optimal control of droplets present in the aerosol expelled while coughing (5, 7).

To conclude, airborne transmission of infectious disease is a major public health concern. Need of the hour is to assess the true contribution of airborne transmission to infection rates so that hospital administrators can implement appropriate control measures to keep respiratory infections under check.

## References

[1] Martin PMV, Martin-Granel E (2006). 2,500-year evolution of the term epidemic. Emerg Infect Dis.

[2] Fiegel J, Clarke R, Edwards DA (2006). Airborne infectious disease and the suppression of pulmonary bioaerosols. Drug Discov Today.

[3] Blachere FM, Lindsley WG, Pearce TA, Anderson SE, Fisher M, Khakoo R (2009). Measurement of airborne influenza virus in a hospital emergency department. Clin Infect Dis.

[4] Writing Committee (CDC, WHO, IUATLD) (1999). Tuberculosis infection control in the era of expanding HIV care and treatment: an addendum to WHO guidelines for the prevention of tuberculosis in heath care facilities in resource limited settings. http://whqlibdoc.who.int/hq/1999/WHO_TB_99.269_ADD_eng.pdf.

[5] Ministry of Health and Family Welfare (2010). Guidelines on airborne infection control in healthcare and other settings. http://www.tbcindia.nic.in/pdfs/Guidelines_on_Airborne_Infection_Control_April2010Provisional.pdf.

[6] Escombe AR, Moore DAJ, Friedland JS, Evans CA, Gilman RH (2007). Natural ventilation for prevention of airborne contagion. PLoS Med.

[7] Zayas G, Chiang MC, Wong E, MacDonald F, Lange CF, Senthilselvan A (2012). Cough aerosol in healthy participants: fundamental knowledge to optimize droplet-spread infectious respiratory disease management. BMC Pulm Med.

